# “Hard to Say, Hard to Understand, Hard to Live”: Possible Associations between Neurologic Language Impairments and Suicide Risk

**DOI:** 10.3390/brainsci11121594

**Published:** 2021-11-30

**Authors:** Alessandra Costanza, Andrea Amerio, Andrea Aguglia, Luca Magnani, Gianluca Serafini, Mario Amore, Roberto Merli, Julia Ambrosetti, Guido Bondolfi, Lisa Marzano, Isabella Berardelli

**Affiliations:** 1 Department of Psychiatry, Faculty of Medicine, University of Geneva (UNIGE), 1211 Geneva, Switzerland; 2 Department of Neuroscience, Rehabilitation, Ophthalmology, Genetics, Maternal and Child Health (DINOGMI), Section of Psychiatry, University of Genoa, 16132 Genoa, Italy; andrea.amerio@unige.it (A.A.); andrea.aguglia@unige.it (A.A.); magnani1991@gmail.com (L.M.); gianluca.serafini@unige.it (G.S.); mario.amore@unige.it (M.A.); 3 IRCCS Ospedale Policlinico San Martino, 16132 Genoa, Italy; 4 Mental Health and Suicide Prevention Center, Department of Mental Health, 13900 Biella, Italy; roberto.merli@aslbi.piemonte.it; 5 Emergency Psychiatric Unit (UAUP), Department of Psychiatry and Department of Emergency, Geneva University Hospitals (HUG), 1211 Geneva, Switzerland; julia.ambrosetti@hcuge.ch; 6 Department of Psychiatry, Service of Liaison Psychiatry and Crisis Intervention (SPLIC), Geneva University Hospitals (HUG), 1211 Geneva, Switzerland; guido.bondolfi@hcuge.ch; 7 Faculty of Science and Technology, Middlesex University, London NW4 4BT, UK; l.marzano@mdx.ac.uk; 8 Department of Neurosciences, Mental Health and Sensory Organs, Suicide Prevention Center, Sant’Andrea Hospital, Sapienza University of Rome, 00185 Rome, Italy; isabella.berardelli@uniroma1.it

**Keywords:** suicide, suicidal behavior, suicidal ideation, suicide attempt, language, aphasia, verbal fluency, prosody, dysprosody, emotions, emotional speech, anxiety, depression, semantic dementia

## Abstract

In clinical practice, patients with language impairments often exhibit suicidal ideation (SI) and suicidal behavior (SB, covering the entire range from suicide attempts, SA, to completed suicides). However, only few studies exist regarding this subject. We conducted a mini-review on the possible associations between neurologic language impairment (on the motor, comprehension, and semantic sides) and SI/SB. Based on the literature review, we hypothesized that language impairments exacerbate psychiatric comorbidities, which, in turn, aggravate language impairments. Patients trapped in this vicious cycle can develop SI/SB. The so-called “affective prosody” provides some relevant insights concerning the interaction between the different language levels and the world of emotions. This hypothesis is illustrated in a clinical presentation, consisting of the case of a 74-year old woman who was admitted to a psychiatric emergency department (ED) after a failed SA. Having suffered an ischemic stroke two years earlier, she suffered from incomplete Broca’s aphasia and dysprosody. She also presented with generalized anxiety and depressive symptoms. We observed that her language impairments were both aggravated by the exacerbations of her anxiety and depressive symptoms. In this patient, who had deficits on the motor side, these exacerbations were triggered by her inability to express herself, her emotional status, and suffering. SI was fluctuant, and—one year after the SA—she completed suicide. Further studies are needed to ascertain possible reciprocal and interacting associations between language impairments, psychiatric comorbidities, and SI/SB. They could enable clinicians to better understand their patient’s specific suffering, as brought on by language impairment, and contribute to the refining of suicide risk detection in this sub-group of affected patients.

*“If you cannot enter the realm of a possible and shared meaning, you are barely able to exist”*

L. Pirandello, 1904. In: Il fu Mattia Pascal. I edition. La Nuova Antologia, Roma.

## 1. Introduction

Suicidal ideation (SI) and suicidal behavior (SB, covering the entire range from suicide attempts, SA, to completed suicides) among patients with language impairment are common in clinical practice (occurring in about one third of these patients [1,2]), especially in acute clinical settings, such as in psychiatric emergency departments (ED), where patients often arrive with both somatic and psychiatric disorders [3,4].

Surprisingly, and with one notable exception [3], we only found individual case reports on this topic in the literature [5,6,7,8,9] (Table 1). Mahgoub and colleagues [6] reported on a woman who was affected by primary progressive aphasia (PPA) and had made an SA in relation to her language impairment. Carota and colleagues [7] described a patient who made an SA in the context of severe Wernicke’s aphasia. The other case reports concern SI and SB in patients with semantic dementia [5,8,9]. Sabodash and colleagues [5] provided an in-detail description of five cases of SI, SA, and completed suicides in patients with semantic dementia in a larger study that compared the suicide risk in patients with semantic dementia (*n* = 25) vs. patients with early-onset Alzheimer’s disease (AD) (*n* = 111). They found 5 patients with SB (including 2 completed suicides) in the semantic dementia group vs. 1 patient with SB in the early onset AD population (*p* < 001) [5]. We did not find any other studies that addressed this topic.

There were some systematic studies which have been repoted [10,11,12,13,14,15,16,17,18,19,20,21] (Table 2), but they approached this topic from a different point of view. Rather than investigating neurological language impairment and its implications as possible SI/SB triggers, they aimed to identify possible deficits in language-related functions, mostly related to verbal fluency, as a part of a broader and more comprehensive profile of executive functions in suicidal vs non-suicidal patients [10,11,12,13,14,15,16,17,18,19,20,21]. In a cross-sectional, case–control study involving inpatients with various clinical diagnoses who had made an SA, Bartfai and colleagues [10] found that patients who had made SA scored significantly lower in verbal fluency when compared to healthy controls. A study that focused on patients with major depressive disorder (MDD) and SA found that patients with MDD and SA had significantly lower scores in letter and category fluency when compared to healthy controls [11]. Investigating whether the presence of SA in MDD patients had an impact on fluency, Keilp and colleagues found a lower fluency in MDD patients with SA when compared to MDD patients without SA [12]. However, later studies by the same lead author found that the inclusion of SA as a covariate did not have any effect on fluency scores [13,14], a finding which was also confirmed in two studies that involved older patients with MDD [15,16]. Examining how the severity of SA impacted on verbal fluency in bipolar patients, Olié and colleagues observed that patients with severe SA outperformed patients with less severe SA regarding to verbal learning, and suggested that the suicidal phenotype may be associated with specific cognitive features, especially in the verbal domain [17]. In a study involving a group of schizophrenic violent offenders, the presence/absence of SA also did not have any impact on verbal fluency [18]. Examining links between verbal fluency and SI/SB, a population-based prospective cohort study involving nearly 5000 older individuals found that poor verbal fluency significantly increased the risk of SI/SB [19]. Near-infrared spectroscopy (NIRS) on suicidal patients while they were being administered verbal fluency tasks has been performed; some studies hypothesized that certain anomalies in the prefrontal cortex might constitute potential biomarkers to identify those patient sub-groups with an increased suicide risk [20,21].

**Table 1:** Case reports on a possible association between suicide risk and neurologic conditions that had led to language impairments.

Ref	Neurological Condition	Cases Summary	SI and SB	Main Findings
[6]	PPA	57-year old woman without previous history of depression. No family history of psychiatric disorders or SI/SB. Symptoms started as difficulty in verbal expression while comprehension of both spoken and written language remained intact. Over the next 6 years, her speech output continued to significantly decrease and her comprehension became delayed. She developed her first episode of depression.	SA by running into traffic	Authors highlighted that the patient had a number of protective factors from suicide (including family support, religion, no chronic medical illness apart from hypertension, and no personal or family history of psychiatric disorders or substance use). They postulated that her debilitating language impairment was a sufficiently severe stressor to lead to SA.
[5]	SD	Patient 1: 63-year old male without personal or family history of mood disorders. No documented current MDD. He presented with progressive difficulty naming objects, loss of comprehension, surface alexia in reading, and prosopagnosia. Patient 2: 62-year old male with a history of depression and SA and possible family history of suicide. He presented with severe impairment on confrontational naming, prominent difficulty with single-word comprehension, surface alexia in reading, and prosopagnosia. He had a diagnosis of semantic dementia. Patient 3: 57-year old female with a lifelong history of depression that worsened with semantic dementia. Her psychiatric history also included periods of hypomania, but no previous SB. No family history of psychiatric disorders. Increased anxiety and depression after semantic dementia diagnosis. She presented with progressive difficulties in word-finding and word-knowing, word generation, and confrontational naming. Prosopagnosia. Patient 4: 60-year old female with a history of depression and SAs. Family history of suicide. Prominent difficulties in word-finding, word-knowing, comprehension, surface alexia, prosopagnosia. Patient 5: 71-year old male, former language teacher, with past and current MDD. No family history of psychiatric diseases. He presented with slowly progressive loss of words, confrontational naming, and inability to understand the meaning of words in different languages. Anomic aphasia.	Patient 1: Recurrent SA, the most recent by drugs abuse Patient 2: Previous and recurrent SA (before the semantic dementia diagnosis). Post semantic dementia diagnosis, intrusive SI and completed suicide by drug overdose. Patient 3: Completed suicide by shooting herself. Patient 4: Prior recurrent SA and current SI with specific plan. Patient 5: A previous SA and current SI with a concrete plan.	Patient 1: The patient stated that he had feelings of hopelessness due to his inability to think because of the loss of words. His impairment preoccupied him to the extent that his conversations were usually focused on his inability. Patient 2: The patient was very distressed and obsessively focused on his loss of words. He stated that he felt “handicapped” by this inability to communicate and comprehend, that this had become a severe burden for his family, and that he wished to die. Patient 3: She had preserved insight and was very distressed; she constantly obsessed over her deficits. Patient 4: She was continually preoccupied by her word loss (“I’m bad with words”) and felt unable to understand what she read (“I have the feeling I won’t live long.”). Patient 5: Authors postulated that in this patient, for whom language competence had been pivotal in his professional life and his feeling of identity, depression was associated with his awareness of language impairment which contributed to his feeling that life was no longer worth living.
[8]	SD	63-year old male admitted to hospital with a depressive condition and presenting with severe anomia and difficulties with semantic knowledge.	Recurrent previous SA	The patient complained of a decreased sense of being human due to the realization that he will not be able to do things in the future that he had done in the past, including linguistic functions and reconstructing autobiographical memory, essential for creating a scaffolding for the future self (“loss of the future self“). This caused hopelessness and depression, leading to SA.
[7]	Severe Wernicke’s aphasia due to ischemic leftperisylvian stroke	66-year old male. No history of mood disorder. Spontaneous language was fluent but uncommunicative due to continuous phonological and verbal paraphasias (jargonophasia). Severely reduced comprehension of auditory, written and visually presented material, naming, repetition, reading, and writing. Finally, the patient was unable to process any kind of communication, even by gesticulation or pantomime.	Survived SA by shooting but became blind as a result.	Even if post-stroke depression and SI are commonly observed, authors postulated here a link between the severe language impairment and SB. They pointed out the difficulty to administer the neuro-psychiatric standardized scale in patients with Wernicke’s aphasia, and emphasized the importance of clinical behavioral observation. They suggested that for every patient with severe Wernicke’s aphasia suicide risk should be seriously considered and carefully explained to the family.
[9]	SD	53-year old male with history of MMD (pre-diagnosis of sematic dementia), although described as non-depressed throughout. He presented with difficulties in single-word semantic comprehension and naming, as well as prosopagnosia.	Previous recurrent SA (during pre-diagnosis MMD and also after diagnosis of semantic dementia). Completed suicide by hanging.	The authors suggested that SB risk was increased in semantic dementia patients, even if depression is absent, and closely related to language impairments and stereotypic behavior characterizingthe semantic dementia. This behavior was considered related to SA made before the onset of semantic dementia.

Legend: MDD = major depressive disorder; OCD = obsessive-compulsive disorder; PPA = primary progressive aphasia; SA = suicide attempt; SB = suicidal behavior; SI = suicidal ideation.

**Table 2:** Studies investigating the neuropsychological profile in suicidal patients (with a particular focus on language-related functions when summarizing their main findings below).

Ref	Study Design	Sample	SI and SB	Main Findings
[10]	Cross-sectional, case-control	Inpatients with various clinical diagnoses who made SA (*n* = 9) vs. non-psychiatric patients (*n* = 15)	SA	Inpatients who made SA had significantly lower scores in verbal fluency compared to controls.
[16]	Cross-sectional, case-control	Older inpatients with MDD and history of SA (*n* = 18) vs older inpatients with MDD without history of SA (*n* = 29)	History of SA	No differences between the two groups in verbal fluency.
[12]	Cross sectional, case-control	Patients with MDD and high-lethality (*n* = 15) or low-lethality (*n* = 14) SA vs. patients with MDD and no SA (*n* = 21)	High- and low-lethality SA	MDD patients having made a high-lethality SA had lower letter and category fluency scores than both MDD patients having made a low-lethality SA and MDD patients not having made SA. MDD patients having made a high-lethality SA had lower category fluency scores than MDD patients without SA. MDD patients with history of SA had lower letter and category fluency scores than healthy controls.
[11]	Cross sectional, case-control	Patients with MDD and history of SA (*n* = 20) vs. Healthy controls (*n* = 20)	History of SA	This finding correlated with blunted increase in perfusion in the prefrontal cortex at SPECT, indicating a possible biological reason for reduced drive and loss of initiative in patients with SA.
[15]	Cross-sectional, case-control	Patients with MDD aged ≥ 65 years with history of SA (*n* = 20) vs patients with MDD aged ≥ 65 years without history of SA (*n* = 20) vs. healthy ≥ 65 years controls (*n* = 20)	History of SA	No significant differences between suicidal and non-suicidal patients with MDD in verbal fluency. Healthy controls had better verbal fluency than suicidal patients with MDD in semantic or phonemic subtest.
[13]	Cross-sectional, case-control	Medication-free patients with MDD and history of SA (*n* = 72) vs medication-free patients with MDD without history of SA (*n* = 80) vs. healthy controls (*n* = 56)	SI and SB	Patients with MDD had lower verbal fluency scores than healthy controls. When past SA was used as a covariate, no difference was observed.
[14]	Cross-sectional, case-control	Patients with history of MDD and SA at various stages of illness (*n* = 80) vs. patients with history of MDD without history of SA at various stages of illness (*n* = 80)	SI and SB	No significant difference in letter and category fluency between patients with history of MDD and SA and patients with history of MDD without SA.
[20]	Cross-sectional, case-control	Patients with MDD and SI (*n* = 31) vs. patients with MDD without SI (*n* = 36)	SI	NIRS performed during a verbal fluency task; hemodynamic changes in the right DLPFC, OFC, and PFC in patients with MDD with SI were significantly smaller than in those without SI. Hemodynamic changes correlated negatively with the severity of SI in DLPFC, OFC, and PFC among patients with MDD.
[17]	Cross-sectional, case-control	Euthymic bipolar outpatients with history of non-severe SA (*n* = 88) vs. euthymic bipolar outpatients with history of severe SA (*n* = 41) vs. euthymic bipolar outpatients without history of SA (*n* = 214)	History of SA	Patients with history of severe SA outperformed patients with history of non-severe SA in verbal learning. Suicidal phenotype may be associated with specific cognitive feature, especially considering verbal domain.
[18]	Cross-sectional, case-control	Patients were violent offenders, grouped into schizophrenia and history of SA (*n* = 26) vs. schizophrenia without history of SA (*n* = 35)	History of SA	No differences between the two groups in verbal fluency.
[19]	Population-based prospective cohort study	4791 older participants	SI, SA, and completed suicides	Poor performances in verbal fluency increased the risk of SI and SB.
[21]	Cross-sectional, case control	Young adults with MDD (*n* = 45) vs. healthy controls (*n* = 32)	SI	NIRS performed during verbal fluency task revealed hypofunction in left dorsolateral PFC, left ventrolateral PFC, and both orbitofrontal cortices in patients with MDD compared to healthy controls.Decreased oxy-HB changes in left ventrolateral PFC corresponded to greater SI in patients with MDD. NIRS may be useful for evaluating SI risk in young adults with MDD.

Legend: DLPFC = dorsolateral prefrontal cortex, FPC = frontopolar cortex, MDD = major depressive disorder, NIRS = near-infrared spectroscopy, OFC = orbitofrontal cortex, oxy-HB = oxyhemoglobin, PFC = prefrontal cortex, SA = suicide attempt, SI = suicidal ideation, SPECT = single photon emission computed tomography.

All these studies were based on a primary hypothesis of the existence of what has been termed “cognitive rigidity”, i.e., the decreasing ability to generate new solutions and escape routes from difficult and seemingly hopeless situations, which has been demonstrated in suicidal individuals and appears to be more pronounced in elderly patients, although it has also been documented in young patients [22,23,24]. This concept is here considered at this stage as a hypothesis and the above-mentioned results appear rather nonconsensual and do not provide any clear evidence to be able to refute or confirm it [25], possibly due to the heterogeneous populations included (individuals with MDD, violent offenders with schizophrenia, healthy controls) and the range of assessment instruments used (psychometric scales, functional neuroimaging).

Collectively, only a small number of reports and studies have examined the possible linkages between language impairments, psychiatric comorbidities, and SI/SB. Moreover, there is a global lack of consistency.

Here, we present a mini-review and describe a case report of a female patient who presented with incomplete Broca’s aphasia, dysprosody, generalized anxiety, depressive symptoms, generalized anxiety, depressive symptoms, who had SI, had made a SA, and later, a complete suicide.

Our literature search employed the following methodology: (i) we searched PubMed/MEDLINE, Scopus, Science Direct, and PsychINFO for relevant articles published between 2 January 2000 until 8 August 2021. We disregarded articles published before 2000, as the diagnostic and research methodologies (psychometric scales, structural and functional neuroimaging, etc.) have changed considerably over the years, which makes comparisons with more dated studies difficult. (ii) Given the heterogeneity in the approaches, in the methods for assessing language impairment (level of production, comprehension, prosody and their interaction with emotional aspects), and the participants’ underlying conditions, we decided to make the review more narrow and focused (mini-), rather than perform a systematic review (according to PRISMA) which would have been difficult, perhaps even futile, and force them into a single all-encompassing review; (iii) to be included in the review, articles had to explicitly mention language difficulties (particularly of a neurological nature) and the suicide phenomenon either in the title or abstract.

Based on the results from our mini-review and this case study, we would like to put forward our central hypothesis that language impairments can exacerbate psychiatric comorbidities, which in turn can aggravate language impairments, creating a vicious cycle in which SI/SB can develop. In the described patient who had a language deficit on the motor side (incomplete Broca’s aphasia and dysprosody), her anxiety, depressive symptoms, and SI were triggered by her inability to express herself, her emotional status, and suffering. 

The aim of this mini-review was to summarise the state-of-the-art. We used the results from the literature review and combined them with a case study to present our above-mentioned central hypothesis, to hopefully raise awareness and elicit further and more dedicated studies, and a more active discussion, in the scientific community.

## 2. Case Presentation

A 74-year old, right-handed female presented with anxiety and depressive symptoms to the psychiatric ED at the University Hospital of Geneva (HUG, Switzerland) in 2012, after SA by abuse of acetaminophen. Although showing depressive symptoms, according to the Diagnostic and Statistical Manual of Mental Disorders (5th ed., DSM-V) [26], the latter could not be classified as a major episode of depression (MDD). She had suffered a circumscribed ischemic stroke two years earlier, which had left her with incomplete Broca’s aphasia and dysprosody. By “incomplete” we mean two things here: (i) the fact that the severity of the speech impairment fluctuated over time, leaving the patient with better abilities on some days and worse on others, and (ii) the fact that the patient always retained some capability to express very simple words and phrases. However, due to the patient’s refusal to undergo testing with psychometric scales, we were unable to assess the severity of this deficit through the use of more objective means, such as battery scores.

The examining physicians, becoming aware of the specific symptoms, retrospectively examined her file (after the consent of the patient and later of her husband), which contained all the clinical, laboratory, and imaging elements that had been collected.

The patient had no family or personal history of psychiatric diseases, nor SI/SB, prior to the onset of her language impairments. Her only other somatic complaint was hypertension. Her family was very supportive and consisted of a husband, three children, and several grand-children. The patient was bilingual in Italian and French and had been working as a writer and translator.

After hospitalization in a psychiatric unit, a cerebral MRI was performed, revealing a diffuse white matter high-signal hyper-intensity in the left posteroinferior portion of the frontal lobe, just anterior to motor cortex. Small white matter high-signal hyper-intensities were also located in the frontal lobe of the right hemisphere. A neuropsychological investigation was not possible because of the patient’s refusal.

The patient was started on psychotropic treatment with the antidepressant sertraline (increasing the dose gradually to 50 mg/day) and, with an anxiolytic purpose, quetiapine (12.5 mg × 3 per day), a drug that with higher posology is mainly prescribed to treat bipolar disorder and schizophrenia. She was treated by a multidisciplinary team of psychiatrists, neurologists, psychologists, and speech therapists. Although the patient showed gradual improvements, both with regard to the anxious and depressive symptomatologies, we observed marked deteriorations in her psychiatric (increased anxiety and depressive symptoms) and neurological patterns (greater difficulty in finding words and more incongruent prosody) whenever she was asked to describe her situation and emotions to the best of her abilities. We also observed a marked improvement in her language expression abilities once her anxiety and depressive symptoms were controlled. The patient described feeling powerless after the onset of her language deficit, which, having been a professional a professional in the field of language, represented an immense and burdensome impairment for her, eventually leading to her SI and SA. The multidisciplinary team worked to offer her other means of expression besides language, and to mobilize her emotions by employing a range of approaches (art therapy, music therapy, psychomotricity, garden therapy, and pet therapy). Therapists attempted to elicit the activation and expression of complex emotional responses by showing her photographs, both generic and of the patient’s family, by engaging the patient in small “role-playing games” that were interesting to the patient, and by encouraging her to use words in songs that she had memorized during her youth, and to which she was emotionally and affectively linked.

While SI was fluctuant initially, after 6 months the patient no longer had any SI and showed an overall improvement. She was therefore discharged and referred to an outpatient facility where she continued treatment for about one year. Then, stating that she was feeling much better, the patient decided to no longer continue her treatment and also wanted to stop attending any sessions with both psychiatrist and speech therapists, contrary to medical advice at the time. Soon after, her anxious and depressive symptoms reappeared, her language expression deteriorated, and, 6 months after stopping her outpatient treatment, the patient performed suicide by drowning in a lake.

## 3. Discussion

In this report, we have described the case of a 74-year old female with incomplete Broca’s aphasia, dysprosody, generalized anxiety, depressive symptoms, and SI/SB. We also performed a mini-review on the possible associations between language impairment (on the motor, comprehension, and semantic sides) and SI/SB (Table 1 and Table 2).

The patient, having been a writer and translator all her life, described her ability to verbally express her actions, thoughts, and emotions to be of crucial importance to her. Additionally, according to accounts by family members, she had always considered language competence both “a part of herself” and “a means of connecting to others”. Losing her language competence on the motor side (Broca’s aphasia and dysprosody), therefore, caused an unbearable suffering to her and led to her psychiatric symptomatology and, eventually, SI/SB.

The association between aphasia and SI has also been observed by Mahgoub and colleagues [6]. They pointed out that different types of aphasia may lead to different levels of suicide risk. For instance, the comprehensive abilities of patients with non-fluent (Broca’s) aphasia are fully intact and their inability to express their thoughts and emotions can therefore lead to the development of intense feelings of frustration. In contrast, patients with fluent (Wernicke’s) aphasia have difficulty comprehending spoken language, and therefore have a limited awareness of their linguistic deficits, which results in lower levels of frustration and SI/SB risk [6,27]. Nevertheless, it has been reported that patients with severe fluent aphasia may also experience intense frustration, leading to SI/SB, [7]. 

Other case reports we found in the literature were focused on patients with semantic dementia. These patients also experienced feelings of frustration, hopelessness and helplessness over the course of a disease that can progressively affect the three aspects of language, namely, the production of words, their comprehension, and the recognition of their meaning [5,8,9]. The realization by a patient of their semantic impairment may cause them to feel “less human” in a world they can no longer “describe”, “read”, and “integrate”, which prevents them from being able to relate to, understand, and belong to that world [28,29,30]. Sabodash and colleagues suggested that suicide risk is increased if semantic dementia is accompanied by depression, insight preservation, and obsessional rumination over the loss of semantic competence [5]. Indeed, a depressive syndrome could be diagnosed in many of these cases and was observed to mediate the association between the type and severity of linguistic impairment and SI/SB risk [6]. However, especially in the elderly with somatic pathologies, there are other, more nuanced situations, such as the one described in our case report, for which a diagnosis of major depressive episode in accordance with the DSM-V criteria [26] is more difficult to establish. Using the definition of “demoralization” by Clarke and Kissane, according to which this psychological condition is precisely described by the presence of hopelessness or disheartenment, a loss of meaning in life, helplessness, a sense of failure, and dysphoria [31,32,33], the “demoralization” construct can also be used to detect suicide risk in these patients [34,35].

In the described patient, Broca’s aphasia and prosody worsened when she was asked to talk about herself, her emotional state, and suffering. Speech prosody typically includes several variables, such as the rhythm, stress, and intonation of speech, which together provide important information beyond the literal word meaning, e.g., clues about attitude or affective state [36]. During the early days of neurolinguistics, the affect or emotions of the speaking subject were treated as para- or extra-linguistic facts, and efforts to understand and describe language were focused on aspects of the code. However, gradually, the independence of these factors in the mechanisms of the communicative system had started to be questioned, allowing room for the subject’s intrapsychic functioning and their interactions with the outside world [36]. The term “emotional speech” has been coined to designate the facets of language which are most related to these latter aspects, of which word production (at the level of motivation/induction to produce them) and prosody would be the preferred vectors and tasks [37].

The relationships between psychic functions and language have been hypothesized especially in the area of prosody, with the coining of the term “affective prosody”, which is, in the medical field, a synonym of “emotional speech”. It is currently accepted that prosody variations in patients with language disorders occur during changes in their mental state, both at an “explicit or propositional” and “implicit” degree [38,39]. A study which investigated post-stroke depression reported that early alterations of affective prosody were associated with a higher risk of post-stroke depression three months following a stroke [40]. Particularly, the more words are congruent with the patients’ concerns, the more significant this interference would be, since these affects would mobilize part of the attentional/executive functions, inducing a double-task situation [41]. The evolution of the patient described in the present report is consistent with the neuro-physio-pathological hypothesis, according to which language is considered to be an agent that receives and integrates the complexity of the cognitive system, deputed to the perception/expression of subjective and intersubjective experience [39].

Studies based on structural and functional neuroimaging have proposed that in right-handed individuals, “affective prosody” would have its neuro-anatomical substrates mainly in the right hemisphere, which is linked with the main and accessory language centers of the left hemisphere via the corpus callosum [38,42,43,44,45,46]. Moreover, an interesting link between “affective prosody” alterations and aphasia severity has been postulated in a sample of stroke patients with left-hemispheric damage, which would represent an informative correlation [47]. The more the circuits concerned are affected by a depressive state, the more the main language centers of the left hemisphere would participate, which reinforces the hypothesis of a significant cooperation between affective/emotional content processing and language, on both the impairment aspects of Broca’s aphasia and prosody [38,47].

Intriguingly, this is in agreement with a recent article by Richard-Devantoy and colleagues [48] which featured the rather poignant title: “Psychological pain and depression: it’s hard to speak when it hurts”. The authors found that high levels of psychological pain were associated with poor phonemic verbal fluency performance in men, even after controlling for confounding factors (age, level of depression, anxiety). The construct of psychological pain appears very similar to Shneidman’s concept of “mental pain”, which has been shown to be strongly associated with suicide risk [49,50]. Some studies have addressed the recovery of these patients, who sought means to alleviate their mental pain, hopelessness, and feelings of despair. Interestingly, Miller and Happel [51], as well as Brown and colleagues [52], showed that participant-generated photographs can aide in the activation and expression of complex emotional constructs, both in patients with schizophrenia [51] and aphasia [52].

**Figure 1:**
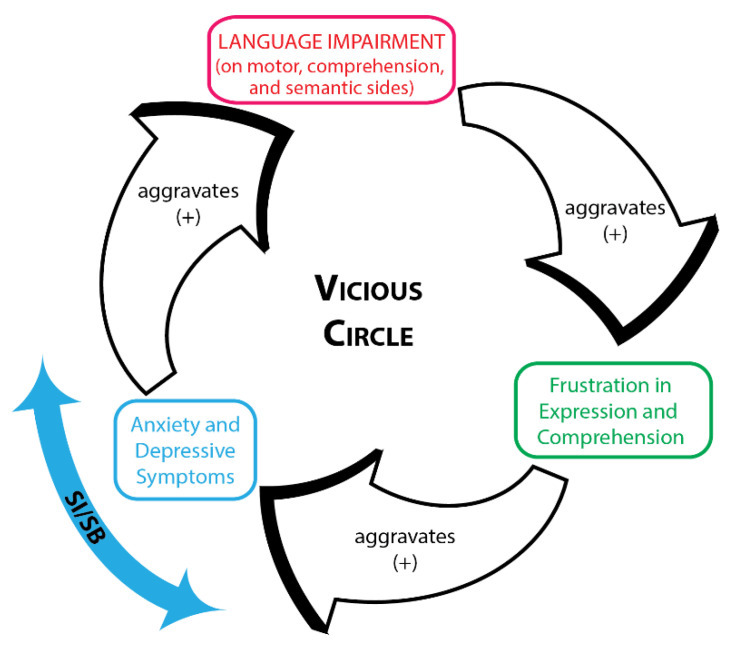
Vicious circle.

Collectively, the findings derived from the case described and the mini-review seem to support our hypothesis of a vicious circle, in which language impairments can exacerbate psychiatric comorbidities, in turn aggravating language impairments and turning affected individuals into what could be termed “desperate mutes”, i.e., creating conditions for the development of SI/SB (Figure 1).

## 4. Limitations

The findings presented in this case report and mini-review should be considered in light of three main limitations. Firstly, the patient was only assessed clinically and by structural neuroimaging, as it was not possible to use psychometric scales or functional neuroimaging, which the patient refused. Secondly, it would have been very helpful and informative to repeat the structural neuroimaging after some months, to better understand the evolution of her language disorders; however, unfortunately, due to the patient’s extreme reluctance to medical examinations, this was not possible. Thirdly, it may not be possible to generalize our particular observations regarding the impact of language impairment on SB/SI, as they are discussed only with regard to other specific case reports and few systematic studies. Fourthly, although the correlative data regarding the hypoactivation of the PFC may suggest a possible connection between verbal fluency and cognitive flexibility, a behavioural assessment of this concept based on verbal fluency alone is somewhat problematic. Lastly, an important aspect that remained open to debate is whether anxiety, depression, and SI/SB are indeed associated with language impairment, i.e., the symptoms of language impairment, or with the underlying disease, e.g., stroke [53,54,55] or dementia [56]. Resulting post-stroke language impairments, particularly on the expressive side, have been observed to be associated with a higher suicide risk (Geriatric Psychiatry Service of Geneva University Hospital, 2008, unpublished observations); in effect, it was postulated that aphasia, which affects about one third of stroke survivors and persists in half the cases, might be one of the factors capable of explaining the post-stroke incidence of SI/SB [57]. Concerning dementias, it is worth pointing out that in semantic dementia, which, together with the behavioral variant of fronto-temporal dementia, FTD (bv-FTD), present the highest suicide risks [58,59], language impairment is one of the hallmark symptoms of the disease. “Affective prosody” is also documented in FTD [60]. However, we did not find sufficient evidence to contribute to this question.

## 5. Practical Implications

Psychopharmaceutical interventions can be considered to reduce the anxiety component. In the presented case, describing an older patient, fractionated, very low-dosage quetiapine was successfully used (exploiting the anxiolytic properties of this drug at a reduced posology, which is otherwise used for other psychiatric disorders). Additionally, Selective Serotonine Reuptake Inhibitors (SSRI) can be useful for this purpose, even if the onset of their therapeutic action is delayed. Complementary, but essential, non-psychopharmaceutical interventions consist of offering other means of expression besides language, and attempting to mobilize emotions by employing a range of approaches (art therapy, music therapy, psychomotricity, garden and pet therapy). Particularly, therapists may try to elicit the activation and expression of complex emotional responses by showing photographs (also patient-generated photographs), both generic and of the patients family, by engaging patients in adapted-to-their-interests, small “role-playing games”, and by encouraging them to use words in memorized songs to which they are emotionally and affectively linked. These strategies are most effective when conducted as part of a multi-disciplinary team, consisting of psychiatrists, psychologists, speech therapists, music therapists, and other rehabilitation therapists.

## 6. Conclusions

Reciprocal and interacting relationships between language impairment, psychiatric comorbidities, and SI/SB frequently occur in clinical practice but have only been sparsely explored from a research perspective. Here, we presented a case report of a female patient who presented with incomplete Broca’s aphasia, dysprosody, generalized anxiety, depressive symptoms, and SB. A mini-review on the possible associations between language impairment (on the motor, comprehension, and semantic sides) and SI/SB was performed. Based on the case report described and findings in the literature, we delineated a vicious circle in which language impairments can exacerbate psychiatric comorbidities which, in turn, aggravate language impairments and create a condition for the development of SI/SB. Patients experiencing severe difficulties with the production and comprehension of language (also of a semantic nature) often display high levels of frustration, and can desire to end their lives due to this burden. Elucidating these associations appears to be relevant, and will enable clinicians to better understand their patient’s specific suffering, as brought on by language impairment, and help refine specific methods for suicide risk detection in these affected patients and outline more comprehensive care approaches that involve psychiatrists, psychologists, speech therapists, and other rehabilitation therapists. A lessons that we learned for the future has been that patients with speech deficits of a neurological origin can provide important insights in informing clinical practice, in addition to with younger patients, who struggle to speak for different reasons, but whom in any case imply interactions between the various levels of language and the world of emotions (the “desperate mutes”). So did Norman and colleagues [61], who recently described on self-harms associated with alexithymia among young adults in their article with the eloquent title “I can’t describe it and they can’t see the rain, an interpretative phenomenological analysis of the experience of self-harm in young adults who report difficulties identifying and describing their feelings”. More systematic studies are needed.

## Data Availability

Materials supporting this case report are available upon request.

## References

[1] Hartlep N.D., Ellis A.L., Pinder S.O. (2013). Rethinking speech and language impairments within fluency-dominated cultures. American Multicultural Studies: Diversity of Race, Ethnicity, Gender and Sexuality.

[2] Costanza A., Ambrosetti J., Wyss K., Bondolfi G., Sarasin F., Khan R.A. (2018). Prévenir le suicide aux urgences : De la «Théorie Interpersonnelle du Suicide» à la *connectedness* [Prevention of suicide at Emergency Room: From the “Interpersonal Theory of Suicide” to the connectedness]. Rev. Med. Suisse.

[3] Ostertag L., Golay F., Dorogi Y., Brovelli S., Bertran M., Cromec I., Van Der Vaeren B., Khan R.A., Costanza A., Wyss K. (2019). The implementation and first insights of the French-speaking Swiss programme for monitoring self-harm. Swiss Med. Wkly..

[4] Costanza A., Mazzola V., Radomska M., Amerio A., Aguglia A., Prada P., Bondolfi G., Sarasin F., Ambrosetti J. (2020). Who Consult an Adult Psychiatric Emergency Department? Pertinence of Admissions and Opportunities for Telepsychiatry. Medicina.

[5] Sabodash V., Mendez M.F., Fong S., Hsiao J.J. (2013). Suicidal behavior in dementia: A special risk in semantic dementia. Am. J. Alzheimers Dis. Other Dement..

[6] Mahgoub N., Avari J., Blau K., Sibel K. (2012). Suicide Risk-Assessment in Patients with Primary Progressive Aphasia. J. Neuropsychiatry Clin. Neurosci..

[7] Carota A., Rimoldi F., Calabrese P. (2016). Wernicke’s aphasia and attempted suicide. Acta Neurol. Belg..

[8] Hsiao J.J., Kaiser N., Fong S.S., Mendez M.F. (2013). Suicidal behavior and loss of the future self in semantic dementia. Cogn. Behav. Neurol..

[9] Kobayashi R., Hayashi H., Tokairin T., Kawakatsu S., Otani K. (2019). Suicide as a result of stereotypic behaviour in a case with semantic dementia. Psychogeriatrics.

[10] Bartfai A., Winborg I.M., Nordström P., Asberg M. (1990). Suicidal behavior and cognitive flexibility: Design and verbal fluency after attempted suicide. Suicide Life Threat Behav..

[11] Audenaert K., Goethals I., Van Laere K., Lahorte P., Brans B., Versijpt J., Vervaet M., Beelaert L., Van Heeringen K., Dierckx R. (2002). SPECT neuropsychological activation procedure with the Verbal Fluency Test in attempted suicide patients. Nucl. Med. Commun..

[12] Keilp J.G., Sackeim H.A., Brodsky B.S., Oquendo M.A., Malone K.M., Mann J.J. (2001). Neuropsychological dysfunction in depressed suicide attempters. Am. J. Psychiatry.

[13] Keilp J.G., Gorlyn M., Russell M., Oquendo M.A., Burke A.K., Harkavy-Friedman J., Mann J.J. (2013). Neuropsychological function and suicidal behavior: Attention control, memory and executive dysfunction in suicide attempt. Psychol. Med..

[14] Keilp J.G., Beers S.R., Burke A.K., Melhem N.M., Oquendo M.A., Brent D.A., Mann J.J. (2014). Neuropsychological deficits in past suicide attempters with varying levels of depression severity. Psychol. Med..

[15] Richard-Devantoy S., Jollant F., Kefi Z., Turecki G., Olié J.P., Annweiler C., Beauchet O., Le Gall D. (2012). Deficit of cognitive inhibition in depressed elderly: A neurocognitive marker of suicidal risk. J. Affect Disord..

[16] King D.A., Conwel Y., Cox C., Henderson R.E., Denning D.G., Caine E.D. (2000). A neuropsychological comparison of depressed suicide attempters and nonattempters. J. Neuropsychiatry Clin. Neurosci..

[17] Olié E., Seyller M., Beziat S., Loftus J., Bellivier F., Bougerol T., Belzeaux R., Azorin J.M., Gard S., Kahn J.P. (2015). Clinical and neuropsychological characteristics of euthymic bipolar patients having a history of severe suicide attempt. Acta Psychiatr. Scand..

[18] Sánchez-Sansegundo M., Portilla-Tamarit I., Rubio-Aparicio M., Albaladejo-Blazquez N., Ruiz-Robledillo N., Ferrer-Cascales R., Zaragoza-Martí A. (2020). Neurocognitive Functioning and Suicidal Behavior in Violent Offenders with Schizophrenia Spectrum Disorders. Diagnostics.

[19] Oh D.J., Han J.W., Bae J.B., Kim T.H., Kwak K.P., Kim B.J., Kim S.G., Kim J.L., Moon S.W., Park J.H. (2021). Executive dysfunction and risk of suicide in older adults: A population-based prospective cohort study. J. Neurol. Neurosurg. Psychiatry.

[20] Pu S., Nakagome K., Yamada T., Yokoyama K., Matsumura H., Yamada S., Sugie T., Miura A., Mitani H., Iwata M. (2015). Suicidal ideation is associated with reduced prefrontal activation during a verbal fluency task in patients with major depressive disorder. J. Affect Disord..

[21] Lee Y.J., Park S.Y., Sung L.Y., Kim J.H., Choi J., Oh K., Hahn S.W. (2021). Reduced left ventrolateral prefrontal cortex activation during verbal fluency tasks is associated with suicidal ideation severity in medication-naïve young adults with major depressive disorder: A functional near-infrared spectroscopy study. Psychiatry Res. Neuroimaging.

[22] Neuringer C. (1964). Rigid Thinking in Suicidal Individuals. J. Consult. Psychol..

[23] Baertschi M., Costanza A., Canuto A., Weber K. (2018). The Function of Personality in Suicidal Ideation from the Perspective of the Interpersonal-Psychological Theory of Suicide. Int. J. Environ. Res. Public Health.

[24] Costanza A., Xekardaki A., Kövari E., Gold G., Bouras C., Giannakopoulos P. (2012). Microvascular burden and Alzheimer-type lesions across the age spectrum. J. Alzheimers Dis..

[25] Huber R.S., Hodgson R., Yurgelun-Todd D.A. (2019). A qualitative systematic review of suicide behavior using the cognitive systems domain of the research domain criteria (RDoC) framework. Psychiatry Res..

[26] American Psychiatric Association (2013). Diagnostic and Statistical Manual of Mental Disorders.

[27] Mendez M.F., Clark D.G., Yudofsky S.C., Hales R.E. (2008). Neuropsychiatric aspects of aphasia and related disorders. The American Psychiatric Publishing Textbook of Neuropsychiatry and Behavioral Neurosciences.

[28] Costanza A., Baertschi M., Weber K., Canuto A. (2015). Maladies neurologiques et suicide: De la neurobiologie au manque d’espoir [Neurological diseases and suicide: From neurobiology to hopelessness]. Rev. Med. Suisse.

[29] Costanza A., Amerio A., Aguglia A., Escelsior A., Serafini G., Berardelli I., Pompili M., Amore M. (2020). When Sick Brain and Hopelessness Meet: Some Aspects of Suicidality in the Neurological Patient. CNS Neurol. Disord. Drug Targets.

[30] Costanza A., Amerio A., Radomska M., Ambrosetti J., Di Marco S., Prelati M., Aguglia A., Serafini G., Amore M., Bondolfi G. (2020). Suicidality Assessment of the Elderly with Physical Illness in the Emergency Department. Front. Psychiatry.

[31] Kissane D.W., Clarke D.M., Street A.F. (2001). Demoralization syndrome—A relevant psychiatric diagnosis for palliative care. J. Palliat. Care.

[32] Clarke D.M., Kissane D.W. (2002). Demoralization: Its phenomenology and importance. Aust. N. Z. J. Psychiatry.

[33] Kissane D.W., Wein S., Love A., Lee X.Q., Kee P.L., Clarke D.M. (2004). The Demoralization Scale: A report of its development and preliminary validation. J. Palliat. Care.

[34] Costanza A., Baertschi M., Richard-Lepouriel H., Weber K., Berardelli I., Pompili M., Canuto A. (2020). Demoralization and Its Relationship with Depression and Hopelessness in Suicidal Patients Attending an Emergency Department. Int. J. Environ. Res. Public Health.

[35] Costanza A., Baertschi M., Richard-Lepouriel H., Weber K., Pompili M., Canuto A. (2020). The Presence and the Search Constructs of Meaning in Life in Suicidal Patients Attending a Psychiatric Emergency Department. Front. Psychiatry.

[36] Drescher M., Tcherkassof A. (2003). La dimension interactive de l’investissement affectif. Les Emotions: Cognition, Langage et Développement.

[37] Perea F., Levivier M. (2012). Nommer/énoncer l’affect. La Lett. De L’enfance Et De L’adolescence.

[38] Wildgruber D., Ackermann H., Kreifelts B., Ethofer T. (2006). Cerebral processing of linguistic and emotional prosody: FMRI studies. Prog. Brain Res..

[39] Tao J., Tan T. (2009). Affective Information Processing.

[40] Villain M., Cosin C., Glize B., Berthoz S., Swendsen J., Sibon I., Mayo W. (2016). Affective Prosody and Depression after Stroke: A Pilot Study. Stroke.

[41] Perlovsky L. (2013). Language and cognition-joint acquisition, dual hierarchy, and emotional prosody. Front Behav. Neurosci..

[42] Schaefer A., Campanella S., Streel E. (2008). La contribution de la neuro-imagerie fonctionnelle à l’étude des emotions humaines. Psychopathologie et Neurosciences: Questions Actuelles de Neuroscinces Cognitives et Affectives.

[43] Ross E.D., Thompson R.D., Yenkosky J. (1997). Lateralization of affective prosody in brain and the callosal integration of hemispheric language functions. Brain Lang..

[44] Ross E.D., Orbelo D.M., Cartwright J., Hansel S., Burgard M., Testa J.A., Buck R. (2001). Affective-prosodyc deficits in schizophrenia: Comparison to schizophrenic symptoms. J. Neurol. Neurosurg. Psychiatry.

[45] Pihan H. (2006). Affective and linguistic processing of speech prosody: DC potential studies. Prog. Brain Res..

[46] Ross E.D., Monnot M. (2008). Neurology of affective prosody and its functional-anatomic organization in right hemisphere. Brain Lang..

[47] Leung J.H., Purdy S.C., Tippett L.J., Leão S.H. (2017). Affective speech prosody perception and production in stroke patients with left-hemispheric damage and healthy controls. Brain Lang..

[48] Richard-Devantoy S., Bertrand J.A., Béziat S., Jaussent I., Cazals A., Ducasse D., Greenway K.T., Guillaume S., Courtet P., Olié E. (2021). Psychological pain and depression: It’s hard to speak when it hurts. Int. J. Psychiatry Clin. Pr..

[49] Shneidman E.S. (1993). Suicide as psychache. J. Nerv. Ment. Dis..

[50] Verrocchio M.C., Carrozzino D., Marchetti D., Andreasson K., Fulcheri M., Bech P. (2016). Mental Pain and Suicide: A Systematic Review of the Literature. Front. Psychiatry.

[51] Miller G., Happell B. (2006). Talking about hope: The use of participant photography. Issues Ment. Health Nurs..

[52] Brown K., Worrall L., Davidson B., Howe T. (2010). Snapshots of success: An insider perspective on living successfully with aphasia. Aphasiology.

[53] Pompili M., Venturini P., Campi S., Seretti M.E., Montebovi F., Lamis D.A., Serafini G., Amore M., Girardi P. (2012). Do stroke patients have an increased risk of developing suicidal ideation or dying by suicide? An overview of the current literature. CNS Neurosci..

[54] Bartoli F., Pompili M., Lillia N., Crocamo C., Salemi G., Clerici M., Carrà G. (2017). Rates and correlates of suicidal ideation among stroke survivors: A meta-analysis. J. Neurol. Neurosurg. Psychiatry.

[55] Pompili M., Venturini P., Lamis D.A., Giordano G., Serafini G., Belvederi Murri M., Amore M., Girardi P. (2015). Suicide in stroke survivors: Epidemiology and prevention. Drugs Aging.

[56] Serafini G., Calcagno P., Lester D., Girardi P., Amore M., Pompili M. (2016). Suicide risk in Alzheimer’s disease: A systematic review. Curr. Alzheimer Res..

[57] Pedersen P.M., Jørgensen H.S., Nakayama H., Raaschou H.O., Olsen T.S. (1995). Aphasia in acute stroke: Incidence, determinants, and recovery. Ann. Neurol..

[58] Haw C., Harwood D., Hawton K. (2009). Dementia and suicidal behavior: A review of the literature. Int. Psychogeriatr..

[59] Purandare N., Voshaar R.C., Rodway C., Bickley H., Burns A., Kapur N. (2009). Suicide in dementia: 9-year national clinical survey in England and Wales. Br. J. Psychiatry.

[60] Leyton C.E., Hillis A.E. (2017). Affective prosody in frontotemporal dementia. Neurology.

[61] Norman H., Marzano L., Oskis A., Coulson M. (2021). “I can’t describe it and they can’t see the rain.” An interpretative phenomenological analysis of the experience of self-harm in young adults who report difficulties identifying and describing their feelings. Curr. Psychol..

